# Understanding the beliefs and attitudes towards mental health problems held by Muslim communities and acceptability of Cognitive Behavioral Therapy as a treatment: systematic review and thematic synthesis

**DOI:** 10.1007/s44192-023-00053-2

**Published:** 2023-11-23

**Authors:** Hind alHarbi, Paul Farrand, Ken Laidlaw

**Affiliations:** 1 Clinical Education, Development and Research (CEDAR), Psychology, Faculty of Health and Life Sciences, University of Exeter, Perry Road, Exeter, EX4 4QG UK; 2 School of Psychology, Faculty of Health and Life Sciences, University of Exeter, Perry Road, Exeter, EX4 4QG UK

**Keywords:** Mental health, Muslim, Help-seeking, Barriers, Beliefs, CBT

## Abstract

**Background:**

Muslims experience the lowest recovery rate from mental health difficulties across all religious groups. The aim of this research is to understand the barriers that prevent Muslims from accessing Cognitive Behavioral Therapy (CBT) and the extent to which these may vary across country of residence.

**Methods:**

Systematic review and thematic synthesis for quantitative, qualitative, and mixed methods studies published in English and Arabic informed by the SPIDER search tool. Methodological quality and risk of bias of included papers were critically appraised independently according to the Mixed Methods Appraisal Tool.

**Results:**

A search of seven databases in the Arabic and English language yielded 3836 studies with 210 studies assessed for eligibility. Employing the Mixed Methods Appraisal Tool resulted in 14 studies included in the thematic synthesis. Seven studies adopted a qualitative methodology employing semi-structured interviews and seven were quantitative descriptive studies.

**Conclusions:**

Muslim communities experience barriers accessing Cognitive Behavioral Therapy at the level of the individual, culture, provider and management. The main barriers were experienced at the individual level which was dominated by the influence of Islam regarding the cause of mental health difficulties, which also influenced the way in which difficulties were managed.

*Systematic review registration*: PROSPERO and registration number: CRD42020192854.

**Supplementary Information:**

The online version contains supplementary material available at 10.1007/s44192-023-00053-2.

## Background

Mental health difficulties are considered a global burden with the World Health Organization estimating that they are experienced by about one in eight people [1]. Based on prevalence identified across several countries, prevalence increases in 2020 with the spread of COVID 19, where anxiety increased by 26% and depression by 28% [2]. Despite increased demands being placed on services, studies have recognized several barriers that reduce the likelihood of mental health help-seeking. General barriers have been identified as related to personal finance, geography, administration, lack of awareness or culture [3–6].

With specific reference to anxiety and depression, research has highlighted barriers at the level of the individual, provider, and system [5]. However, whilst a range of barriers have been identified at different levels, religion was only considered as a part of culture which itself was only considered within the individual level [5]. This fails to recognize the importance that religion serves as one of the pillars of culture that may have a significant impact on the response of the individual to psychological treatment in general [7].

### Muslims mental health

Failing to consider the influence that religion has upon mental health help-seeking may in part account for some of the variations in access and recovery related to ethnic diversity. For example, with respect to the Improving Access to Psychological Therapies (IAPT) programme implemented across England for the treatment of depression and anxiety, members of the Black, Asian and Minority Ethic population continue to experience a disparity compared to white British [8]. With respect to religion alone, such variations range from a recovery rate of 54.5% for followers of Jainism to the lowest recovery rate of 40.3% for followers of Islam [9, 10].

### Barriers to access mental health services

Poor access rates for Muslims may reflect the strong relationship they have with their Islamic religion that influences the way people from the community interpret what they are exposed to in life [11]. With respect to mental health difficulties, people from Muslim communities may interpret them through aspects of Islam such as reflecting evidence of ‘weakness of faith’ or as a punishment from Allah for incomplete religion or sinful acts [12–14, 16]. Furthermore, Muslims may interpret mental difficulties as related to ‘evil eye’, ‘Jinn’ possession or black magic [15]. Hoverer, the stronger a Muslims is in their cultural and Islamic beliefs of psychological problems, the less likely they are to seek mental health services [16].

### Muslims sects

Although all Muslim communities agree on the worship of Allah and recognize the pillars of Islam, they differ in their interpretation of Islamic law (Sharia) [17]. Such differences can vary between Muslim sects (e.g. Sunni, Shi’a, Sufism) and furthermore for linguistic, political, or cultural reasons that may differ by country of residence [17]. Barriers that prevent Muslims from accessing services for the treatment of mental health difficulties may therefore vary. Understanding the extent that such barriers alongside the acceptability of evidence-based psychological therapies vary is therefore of importance [18, 19].

This systematic review and thematic synthesis aim to gain a further understanding regarding the barriers that prevent members of the Muslim community accessing evidence-based psychological therapy for the treatment of mental health difficulties. Consideration will be given towards the extent that barriers may vary across country of residence and furthermore towards the acceptability of Cognitive Behavioral Therapy (CBT) as a treatment.

### The systematic review aim

To understand barriers that prevent adult Muslims from accessing mental health services with comparisons undertaken between Muslim communities resident in different countries.

### Review questions

With respect to adult Muslim communities:

- What is the understanding of mental health?
  - What are the beliefs and attitudes regarding causes and treatment of mental health difficulties?
  - Do beliefs held regarding mental health and treatment vary?
  - Do barriers accessing mental health treatment exist and vary?
  - Is CBT acceptable for the treatment of mental health difficulties?

## Methods

### Design

This systematic review and thematic synthesis was undertaken with strict adherence to the published protocol [20] and registered with PROSPERO (Registration No. CRD4202019285).

### Search strategy

The search was undertaken on the following research databases: PubMed/MEDLINE, CINAHL, PsycINFO, Ovid MEDLINE, Embase and the Index Islamicus religious database for studies published in the English language. Furthermore, the Saudi Digital Library (SDL) was searched for publications in the Arabic language. The search was start in July 2020, and was completed in October 2020.

### Eligibility criteria

Papers written in English or Arabic from 1980, published in peer-review journals and meeting review-specific eligibility criteria informed by the SPIDER search tool [21]. Grey literature and unpublished papers, book chapters, conference papers or dissertations were excluded. Studies focusing on general barriers have not been limited to any specific psychological intervention. Studies that focused on barriers to accessing a specific psychological therapy were limited to CBT. Full study Inclusion and Exclusion Criteria are reported in Additional file 1.

### Study selection

All titles and abstracts of papers written in English were screened by HaH with the full text of articles included from the title and abstract screen independently reviewed by HaH and PF. Screening of Arabic studies was undertaken solely by HaH. Quality assessment was undertaken by HaH and PF independently. Any differences at screening or quality assessment were resolved by wider discussion with KL to reach consensus.

### Analysis

Thematic synthesis [22] was used to support interpretation of data obtained from qualitative, quantitative, and mixed methods research beyond that undertaken by authors of included studies. Results are presented (Additional file 2) according to the Preferred Reporting Items for Systematic Reviews and Meta-Analyses (PRISMA) guidelines [24]. To ensure rigor HaH coded each line of identified studies with separate coding by PF. Analysis was reviewed with consensus reached with PF and KL.

Three stages of thematic synthesis were undertaken.

- First Stage: Text coding. To support data organization, data from the 14 included studies were entered verbatim into NVivo 12. HaH coded each line of these studies with separate coding by PF.
- Second Stage: Developing descriptive themes. Codes were organized based on similarities and differences to establish a hierarchical tree structure and grouped to represent identified themes [22].
- Third Stage: Generating analytical themes. All descriptive themes were reviewed to generate analytical themes to address the objective of the review to gain an understanding MHDs in Muslim communities. However, given the overlap between Cultural barriers and Islamic barriers, discussions with PF and KL were undertaken. Consensus was reached whereby any barrier that have evidence from the Qur’an and Sunnah (Prophet Muhammad says) represented an Islamic belief. Whereas if the barrier was not evident it was considered to represent a cultural barrier [11].

### Risk of bias of individual studies

Informed by the Mixed Methods Appraisal Tool (MMAT) [23], methodological quality and risk of bias associated with included papers was critically appraised independently by HaH and PF.

### Reflexivity

The principal researcher (HaH) was a Muslim working as Clinical Psychologist at King Saud Medical City in Saudi Arabia, with an understanding of Muslim beliefs and attitudes held towards mental health problems. The rest of the team (PF and KL) were non-Muslim researcher from the University of Exeter, UK, with no prior experience with Muslim communities. Being a Sunni Muslim, the potential that HaH may interpret the analysis through her own Islamic beliefs was recognized. During each stage of analysis therefore, frequent meetings were held with PF and KL to question and justify derived themes and sub-themes.

## Results

**Fig. 1:**
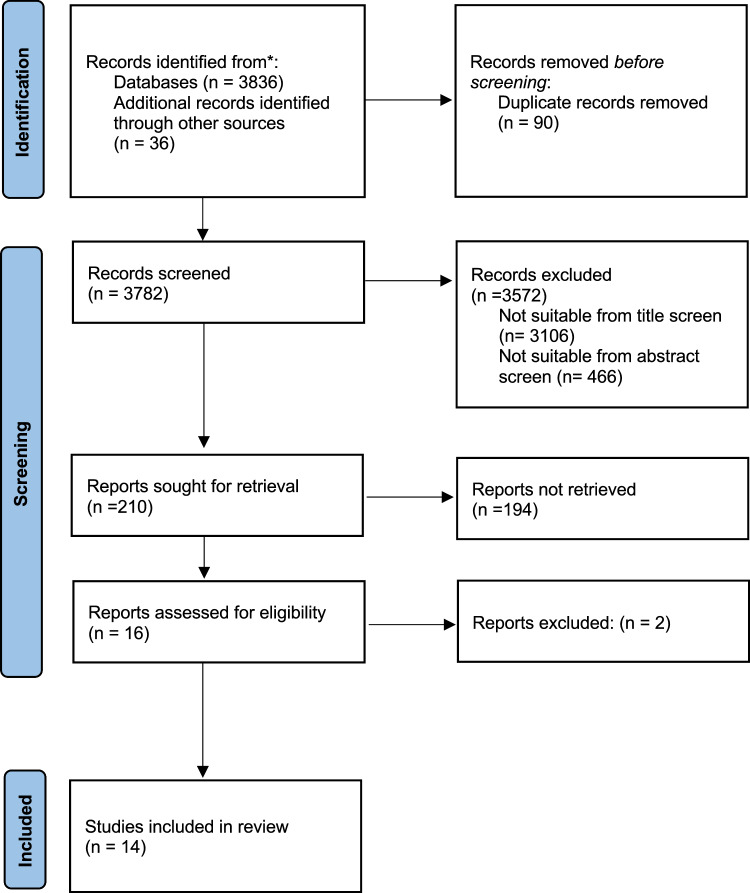
PRISMA flow diagram [24]

The following databases yielded 3836 studies published between 1980 and 2020; SLD (n = 2330); PsychInfo (n = 1310); EMBASE (n = 69); Pub Med (n = 66); Medline (n = 52) CINAHL (n = 8) and Index Islamicus (n = 1). Other reference lists and citation checks identified an additional 36 studies. After duplicates were removed, a total number of 3782 studies were identified for screening which was reduced to 210 that were assessed for eligibility following a title and abstract review removing studies that did not meet inclusion criteria (Fig. 1). Of the 210 studies assessed for eligibility, 194 were excluded.

### Quality assessment

The MMAT [23] was used to appraise the quality of16 studies included in the Quality Assessment. Given a poor standard of reporting the method, two studies were excluded at this stage resulting in the final analysis undertaken on 14 studies (Table 1). Applying the MMAT highlighted the qualitative studies to be of higher quality than the quantitative studies. In terms of the individual components, all qualitative studies reported outcome data and clearly described study procedures. Alternatively, in the quantitative descriptive studies the sample was not representative of the target population [35, 36, 39], or response bias was not reported [36, 38].

**Table 1:** Risk of bias table for qualitative and quantitative studies

Study	Screening questions		1. Qualitative studies					4. Quantitative descriptive studies				
	S1. Are there clear research questions?	S2. Do the collected data allow to address the research questions?	1.1. Is the qualitative approach appropriate to answer the research question?	1.2. Are the qualitative data collection methods adequate to address the research question?	1.3. Are the findings adequately derived from the data?	1.4. Is the interpretation of results sufficiently substantiated by data?	1.5. Is there coherence between qualitative data sources, collection, analysis and interpretation?	4.1. Is the sampling strategy relevant to address the research question?	4.2. Is the sample representative of the target population?	4.3. Are the measurements appropriate?	4.4. Is the risk of nonresponse bias low?	4.5. Is the statistical analysis appropriate to answer the research question?
[25]	Yes	Yes	Yes	Yes	Yes	Yes	Yes					
[26]	Yes	Yes	Yes	Yes	Yes	Yes	Yes					
[27]	Yes	Yes	Yes	Yes	Yes	Yes	Yes					
[28]	No	No										
[29]	Yes	Yes	Yes	Yes	Yes	No	No					
[30]	No	No										
[31]	Yes	Yes	Yes	Yes	Yes	Yes	Yes					
[32]	Yes	Yes	Yes	Yes	Yes	Yes	Yes					
[33]	Yes	Yes	Yes	Yes	Yes	Yes	Yes					
[34]	Yes	Yes						Yes	Yes	Yes	Yes	Yes
[35]	Yes	Yes						Yes	No	Yes	Yes	Yes
[36]	Yes	Yes						Yes	No	Yes	No	Yes
[37]	Yes	Yes						Yes	Yes	Yes	Yes	Yes
[38]	Yes	Yes						Yes	Yes	No	No	Yes
[39]	Yes	Yes						Yes	No	Yes	Cannot tell	Yes
[40]	Yes	Yes						Yes	Yes	Yes	Cannot tell	Yes

### Study characteristics

Of the 14 studies included in the thematic synthesis, seven adopted a qualitative methodology employing semi-structured interviews alongside seven quantitative descriptive studies (Table 2).

**Table 2:** Characteristics of included studies

References	Aim	Population (number)	Mental health difficulty	Setting	Comment
*Qualitative studies*					
[25]	Gather information to culturally adapt CBT for depression and anxiety in KSA* and Bahrain	42 Patients (Bahrain 24; KSA 18). 11 Caregivers (Bahrain 7; KSA 4). 16 Psychiatrists and psychologists (Bahrain 11; KSA 5)	Depression and Anxiety	KSA and Bahrain	Only patient sample used
[26]	Explore the role of religion in the experience of OCD among young Saudi females	15 Female	OCD	KSA	
[27]	Identify differences in beliefs about mental illness and explore the extent religious beliefs and practices impact on beliefs about causes of, and treatments for, mental illness	52 Female (Pakistani Muslin 13; White Jewish 13; White Catholic, 10; Indian Hindu, 9; Black African Christian, 7)	Depression and Schizophrenia	UK	Only Pakistani Muslin sample used
[29]	Compare perceived effectiveness of religious help with other forms of help	59 Female	Depression and Schizophrenia	UK	Only Pakistani Muslim sample used
[31]	Develop guidelines for adapting CBT for Schizophrenia in Pakistan	33 Patients; 30 Carers; 15 Psychiatrists; 14 Psychologists	Schizophrenia	Pakistani	Only patient sample used
[32]	Investigate attitudes of Pakistani families living in the UK towards mental health issues and mental health services	29 First generation Females resident in the UK; 23 Second generation Females; 22 Males	N/A	Pakistani in UK	
[33]	Explore Muslims understanding of mental health	14 Muslims	N/A	UK	
*Quantitative studies*					
[34]	Identify factors contributing to the refusal of psychotherapy	143 Patients	N/A	KSA	
[35]	Examine the influence of social factors on person's attitude towards people with mental illness	231 Public; 173 medical students; 64 relatives	N/A	Oman	Only public sample used
[36]	Examine attitudes toward seeking psychological help regarding self-esteem and depression	350 College students (273 females; 53 males, 24 not specify gender)	Depression	Emirate	
[37]	Measure beliefs of Arabic primary care patients about causes of their physical symptoms to quantify beliefs consulting GPs in Saudi Arabia and examine if patients with psychological problems differ from others in religious and supernatural beliefs	224 Primary care patients	N/A	KSA	
[38]	Identify the role of popular therapy and its effects, treatment methods and the degree to which patients with mental health difficulties accept it	150 Patients from psychiatric clinic in Riyadh	N/A	KSA	
[39]	Explore Muslims’ beliefs about Jinn, black magic and the evil eye as a cause of mental health problems and whether doctors, religious figures or both can provide treatment	111 Muslims (Male, 59; Female, 52)	N/A	UK	
[40]	Identify beliefs regarding causes and treatment of auditory hallucinations, and levels of social acceptance in a *KSA and UK sample	Patients (281: KSA, 150; UK, 131)	N/A	UK and KSA	Only KSA sample used

### Analysis

Three stages of thematic synthesis were undertaken. In the first stage (Text coding), 13 initial codes were repeated more than once, and 12 sub-codes. In Second Stage (Developing descriptive themes), Following discussion, HaH and PF discussed the codes and sub-codes to derive brief descriptions (Table 3).

**Table 3:** Description of codes and sub cod

Codes	Sub codes	Description
Cultural sensitivity		Knowledge of cultural differences between Islamic and non-Islamic cultures
Diagnosis		Problems diagnosing MHDs
Family Role		Belief a person will only find support for MHDs within the family
Fear of Stigma		Attempting to hide or reject mental illnesses to avoid society's stigma regarding MHDs
Islamic beliefs about causes of MHDs	Evil eye	Belief people have ability to harm others just by looking at them
	Jinn	Belief a person is possessed by an invisible alien spirit or other para-human force that can control actions
	Punishment from Allah	MHDs considered to be a punishment from Allah for sins a person commits
	Strong Faith	Belief having a strong faith will prevent a MHD
	Test from Allah	Belief MHDs represents an affliction from Allah to test the depth of faith
Language		Language differences between the therapist and patient
Psychotherapy	Therapists Religion	Religion of therapist and effect of this on acceptance as a therapist
	Trust in Therapist	Confidence that a mental health professional is able to provide assistance
Religious	Faith Based Healer	Belief MHDs can be resolved by healers using Quran recitation and supplication
	Refer to Islam	The application of the true principles of Islam considered to be the best ways to manage MHDs
	Religious Acts	Religious acts (such as praying and reading the Qur’an) believed to represent an important part of dealing with MHDs
Service Awareness		Having awareness regarding the availability of mental health services and ease of access to them
Status of Muslim Women		Dependence of woman on men to undertake certain activities
Western beliefs about causes of MHDs	Biological Basis	Beliefs highlighting that MHDs have a biological basis
	Life Influences	Difficult life events serve as a precursor to a MHD

**Fig. 2:**
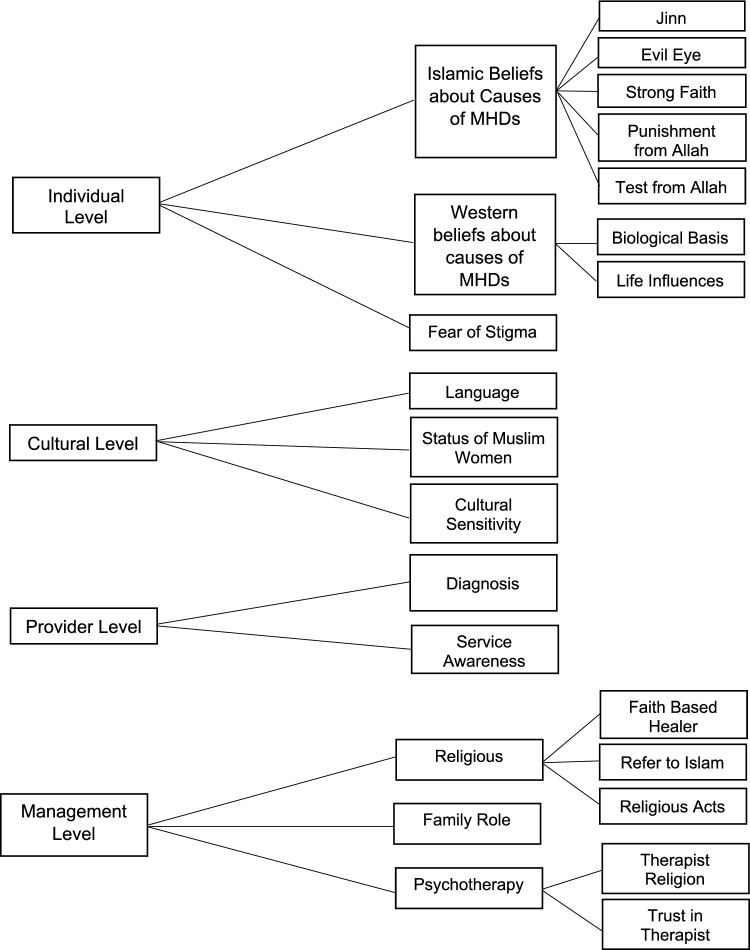
Hierarchical tree structure [24]

In third Stage: (Generating analytical themes), After repeated examination of the descriptive themes, four main themes were identified (Fig. 2).

### Barriers accessing mental health services in Muslim communities

These were identified as occurring at four main levels repeated across the majority of studies included within this review.

### Individual level

Related to the impact of personal beliefs and faith have on the interpretation and understanding of mental health difficulties. These included Islamic beliefs about the causes of mental health difficulties, western beliefs about the causes of mental health difficulties and fear of stigma. Beliefs associated with the Islamic religion about the causes of mental health difficulties were mentioned regardless of whether people considered themselves ‘good Muslims’ or not [33]. Such Islamic beliefs led the person to go to Islamic religious therapists rather than access mental health treatment [26].

The most repeated Islamic belief related to the cause of Mental Health Difficulties was the belief in Jinn [25, 26, 32, 33, 35, 37–40]. This belief represented a strong reason to justify the presence of mental health difficulties across all Muslims communities. However, the extent to which it was held varied between 80% of UK Muslims [39] and 17% of Saudi Muslims [38]. Studies based on a Saudi sample highlighted that belief in Jinn was not as strong as expected [25, 37]. Additionally, within study samples an association between Evil Eye and mental health difficulties [26, 31, 37–39] was reported by 73% of UK Muslims [39] and again by a much smaller number of Saudi Muslims (38%) [38].

Of significance however, participants in one study [26] believed that rather than directly causing mental health difficulties, Evil Eye triggered a biological imbalance. Experiencing a mental health difficulty was also recognised as being a punishment from Allah [38], and as an affliction to test a person’s patience in their life [33, 37, 40]. In addition, belief that a mental health difficulty afflicts a person because of a weak faith [26, 27, 33] have identified in this level, Muslims believe that people of strong faith are not experienced mental health difficulties [26, 33]. On occasions, the strength of belief in Islamic causes resulted in several UK Muslims participants considering the request for psychological help as representing a direct rejection of Allah [33].

Whilst Islamic beliefs were pervasive amongst many participants across studies however, beliefs attributing mental health difficulties to having a biological basis or arising through life influence were also highlighted [25–27, 31, 34, 37]. On occasions, some participants from Pakistan were able to integrate these explanations with Islamic beliefs. However, even when recognized, placing an emphasis on the necessity of respecting religious beliefs was maintained [31].

As in most cultures, stigma directed towards people with mental health difficulties was evident across all Muslim communities [27, 32, 33, 35, 36, 40]. In Muslim communities however, stigma was identified to arise from Islamic beliefs where mental health difficulties are considered as evidence regarding a weakness of faith [27]. Stigma also arose from concerns that an entire family would be viewed negatively in the event that any member was experiencing a mental health difficulty. Refusing to admit a family member was experiencing a mental health difficulty was therefore not uncommon within families and thereby prevented help-seeking [32]. Alternatively, one study examining the beliefs held by an Emirate University student population towards the causes of mental health difficulties highlighted a greater acceptance of seeking mental health services with lower levels of stigma [36]. This indicates that stigma may be less prevalent in Muslims with a higher level of education [40].

### Cultural level

Represents a range of sub themes specifically related to the Islamic communities which includes Language, State of Muslim Women and Cultural Sensitivity.

Language represents an especially important barrier to accessing mental health services in multi-lingual countries. Specific difficulties were related to a lack of translated information regarding ways to access mental health services [32, 33]. Furthermore, State of Muslim Women was identified with respect to different expectations and behavioral freedoms placed on men and women. Of particular significance were cultural restrictions placed on women receiving treatment from a male therapist when they were not accompanied [26, 32, 34]. The inability of women to attend treatment on their own hinders their access to psychological therapy [34] and thereby prevents them from making their own treatment choices [26].

Cultural Sensitivity represents one of the most important barriers at the cultural level [32–34, 36]. This barrier was reflected across studies in several ways within the Muslim community, such as having an appreciation that people from Saudi Arabia and Emirate prefer to talk about their personal matters in a general way that avoids specific detail [34, 36]. Placing an emphasis on a detailed assessment to inform diagnosis and treatment may therefore prevent many Muslims seeking treatment from a mental health service [34, 36]. Within one study, 56% of the Saudi study sample reported being unwilling to accept psychotherapy given a refusal for interpersonal openness [34].

Differences between males and females should also be taken into consideration regarding help-seeking within Muslim communities [32, 34]. In Muslim communities, males and females are separated from early childhood in schools and later in the workplace and many public places [12, 15]. Factors may therefore appear in psychological treatment, such as sensitivity to dealing with a female patient if the therapist is male. This makes the application of psychological treatment techniques such as relaxation unacceptable [34], which was evident across Muslim communities in different countries [32–34].

### Provider level

Represents the relationship between the person and the mental health service which includes features associated with diagnosis and service awareness. Studies on UK Muslims highlighted that physical symptoms were the most significant reason for them to seek treatment for a mental health difficulty [33]. This often makes the GP the first contact for people seeking treatment [25, 32, 33, 36, 37]. Furthermore, several studies also highlighted having a poor understanding regarding the availability of mental health services or did not have enough information about how to access them. Lack of information served as a help-seeking barrier for Muslims in the UK, Emirate and Saudi Arabia [33, 34, 36].

### Management level

Reflects the way that mental health difficulties are managed by members of the Muslim community [26, 27, 29, 31–33, 38–40]. Consistent with beliefs that Islamic religion is a main cause of mental health difficulties, most studies highlighted that religious healers or acts had a seminal role in management. Consequently, the role of mental health services in the treatment of mental health difficulties was identified in far fewer studies [25–27, 31, 33, 34, 39]. Many studies [26, 27, 29, 31, 33, 38–40] highlighted that senior authority figures in Islam, such as the Hakim or Sheikh, are often initially approached to treat mental health difficulties by members of the Muslim communities in Saudi Arabia, UK, and Pakistan. However, in two studies [26, 31], all Saudi and Pakistani participants sought psychological help whilst still going to religious therapists. Some patients were also reported as consulting more than one religious healer before trying psychotherapy [31].

However, many Muslims prefer to engage in religious acts on their own [25–27, 29, 33, 36–38, 40], rather than go to a religious healer [26]. Common religious acts undertaken to manage mental health difficulties included Holy Quranic recitation [25, 27, 36, 38], prayer [25–27, 29, 33, 40], supplication [33] and Wudu, where certain parts of the body are washed before praying [33]. Where mental health difficulties are considered to arise as a Weakness of Faith, trying to return to true Islam to strengthen faith was recognized as an important way to manage mental health difficulties [29, 33]. Referring to Islam was highlighted as providing psychological relief by showing submission to Allah [33].

In addition to religious involvement, the family was considered to represent a major source of management for mental health difficulties by representing a source of psychological support and assisting with problem solving [25–27, 32, 33, 36]. However, two studies highlighted occasions where the family was viewed negatively with respect to mental health difficulties [25, 33]. Within Saudi Arabia and Bahrain, the family was recognized as a major cause of mental health difficulties through tense relations and social pressure exerted on its members [25], whilst also recognized as a source of poor advice in the UK [33].

Several studies specifically referred to the acceptance of psychotherapy in Muslim communities in Saudi Arabia, Bahrain, UK, Pakistan and Emirate [25–27, 31–34, 36, 39]. Over half of the Pakistani participants [31] were aware of psychological treatment and expressed their wish to receive it. Additionally, awareness regarding the availability of CBT and information found on social media was highlighted [25].

However, several considerations are necessary for psychotherapy to be found acceptable for Muslim communities. Several studies focusing on UK Muslims indicated that requiring the therapist to be of the Islamic religion was important [27, 39]. In communities where Muslims are considered a minority and most likely to be referred to a non-Muslim professional, it was believed that therapists of the same religion would be better placed to understand patient attitudes towards mental health difficulties [27, 33, 39]. It was also considered that having a better understanding regarding Islam would enable the therapist to address religious beliefs regarding treatment and highlight religious acts that may be of comfort alongside providing psychological therapy [27, 33, 39]. Whilst there was a strong preference for a Muslim therapist however, one study of UK Muslims indicated that therapist experience rather than having Islamic faith was of greatest importance [27]. Several participants also mentioned that a therapist of the same religion could have both advantages with respect to a greater cultural understanding, alongside concerns that details of the consultation could be divulged to the Muslim community [27].

## Discussion

This review examined barriers accessing mental health services and attitudes concerning mental health difficulties in adult Muslims. Thematic synthesis highlighted a significant influence of religious beliefs and Islamic teaching on Muslim attitudes regarding the causes of mental health difficulties. In particular, all studies included in this review showed cultural and religious factors were essential in shaping attitudes and understanding regarding mental health difficulties and treatment preferences [25–27, 29, 31–41]. This indicates the need for these factors to be considered when addressing major help-seeking barriers experienced by Muslims accessing mental health services [41].

Barriers coalesced at the level of the individual, cultural, provider and management. In comparison with previous research regarding the recognition of individual and cultural barriers [5, 18], important differences arose regarding Systemic barriers. Whilst in the current review, Systemic level barriers were not identified. Furthermore, differences arose with recognition of cultural factors as representing a distinct barrier in this review. This potentially reflects a focus in previous studies [e.g., 5] on barriers experienced by people with a white ethnic background. Whereas studies focusing on people from other ethnicities have also highlighted a seminal importance of cultural barriers [18, 41]. With respect to Muslim communities, the Islamic culture is recognized as influencing all aspects of life [42].

Beliefs regarding the cause of mental health difficulties held by different Muslim communities was recognised as a major individual level barrier. For example, Muslims may interpret mental health difficulties as being determined by ‘Evil Eye’, ‘black magic’ or ‘Jinn possession’ [11, 15]. Such explanations are acceptable to members of the Muslim community as they are derived from the Quranic evidence and sayings of the Prophet Muhammad that confirm the existence of these unseen forces [13, 25]. In addition to the existence of religious evidence for these beliefs, they may also represent acceptable explanations for mental health difficulties as they are considered to remove personal responsibility [26].

Furthermore, belief in ‘Evil Eye’ may represent an acceptable explanation given those that attract ‘Evil Eye’ are considered to have positive attributes from which they draw esteem [26, 37]. However, caution should be taken before assuming all Muslims share such beliefs. To a much lesser extent, conventional Western beliefs identifying factors such as life influences and biological factors causing mental health difficulties were also recognized [25–28, 31, 33, 34]. Islamic beliefs are therefore not held by all Muslims as representing the cause of mental health difficulties. This reinforces the importance that practitioners maintain a patient-centered approach when working with patients regardless of ethnic or religious background [43].

The influence of cultural and religious factors also appeared in management level help-seeking barriers that highlighted a preference for religious treatment over psychotherapy. This highlights the seminal importance of the Islamic faith whereby mental health difficulties are directly attributed to religion. This potentially helps to account for why many Muslims fail to seek psychological therapy [44]. Religious healers in Muslim communities therefore often represent the first step for treating a mental health difficulty [45]. On occasions when help-seeking for psychological therapy is undertaken it is largely sought to complement religious treatment [11]. Combining evidence-based psychological therapy with features of the Islamic faith may therefore be of significant benefit given that Muslims are able to work with two explanatory models simultaneously [46]. At the same time, according to Islamic sources, there is no contradiction between combining modern treatment (as psychological treatment) and Islamic treatment [11].

Whilst many studies included within the systematic review identified the role of religion as influencing beliefs and attitudes held towards mental health difficulties, no study considered how barriers may differ between Muslim communities resident in different countries. Failing to recognize these differences makes addressing individual level barriers problematic. Although Muslim communities share the same religion, they differ according to Islamic sect and local culture [47].

Differences between Muslim communities did however emerge with respect to the role of the family. All communities recognized the importance of the family as a primary source of support for mental health difficulties [26, 27, 33, 36]. However, within Saudi and Bahraini communities, the family was also seen as one of the causes of mental health difficulties [25]. Potentially however, differences regarding the influence of the family on mental health difficulties arose because the study only recruited participants in large cities in Saudi Arabia (Dammam) and Bahrain (Manama). In comparison to family’s resident in villages and small cities, the family has become more individualistic and independent within larger cities [48].

Further differences between Islamic communities arose regarding the impact of stigma. Amongst most communities identified in this review, fear of stigma was highlighted as a major barrier that prevented communities from seeking help. However, participants from the Emirati community were more likely to accept the idea of seeking psychological help [36]. It is noteworthy however, that the sample in this study was of Muslim University students. Therefore, the extent to which differences may be dependent on the Emirati community or level of education is unclear.

### Directions for future research

The most significant area in need of further research addressing barriers to accessing mental health services is associated with recognizing the extent to which beliefs regarding mental health difficulties vary by Muslim communities resident in different countries or by Islamic. Current research largely fails to discriminate between these areas and consequently the extent to which attempts to overcome barriers can be generalized is unknown.

Furthermore, there are only two studies [25, 31], that have examined cultural adaptations that may enhance the acceptability of CBT for Muslims, with these studies focusing on the attitudes of professionals rather than patients. Understanding ways to enhance acceptability is important given that amongst many Muslims, CBT may be sought to complement rather than replace religious treatment [11]. Examining barriers accessing psychological therapy that arise within the family given it significance within Muslim communities and identifying ways to address these barriers also represents areas for future research identified in this systematic review.

### Limitations

Transferability of results across Muslim communities resident in different countries may be limited given that studies were restricted to those published in English or Arabic whilst many studies of the Muslim community may have been published in other languages. Another limitation is that the main targeted population in this review comprised adult Muslims that have not received psychological therapies for the treatment of mental health difficulties as well as patients with experience of CBT treatment. Finally, only published studies were included in this review which highlights the potential forpublication bias [49].

### Clinical implications

To enhance acceptability the most important adaptation to CBT is associated with the need to acknowledge Islamic beliefs regarding mental health and integrate these into the psychological intervention [25, 33]. Furthermore, there is a need to educate non-Muslim practitioners regarding the importance of Islamic beliefs in the lives of Muslims and the extent they inform their interpretation of mental health difficulties. However, whilst the acceptability of CBT can be enhanced through cultural adaptations, on many occasions it may be sought to complement religious treatment [11]. Therefore, to promote help-seeking, it may be helpful for service providers to engage with religious leaders [13, 47].

## Conclusion

It is possible to apply CBT in Islamic communities, however adaptations are first required before implementing into service delivery [25, 31, 33]. These can help address individual, cultural, provider and management level barriers that were identified as important in preventing Muslims from accessing mental health services. Religious beliefs are of significant importance when explaining mental health difficulties and regarding the impact of these beliefs on seeking treatment. However, Muslim communities that can vary by sect and country of residence may vary in the extent to which they respond to adaptations and different help-seeking barriers may be evident. Gaining a better understanding of cultural level barriers and the extent they vary across communities may therefore be significantly important when developing psychological interventions for Muslims.

### Supplementary Information

Below is the link to the electronic supplementary material.

Supplementary file 1 (PDF 389 KB)

Supplementary file 2 (PDF 58 KB)

## Data Availability

Data sharing is not applicable to this article as no datasets were generated or analyzed during the current study.

## Abbreviations

CBT: Cognitive behavior therapy
MHDs: Mental health difficulties
MMAT: Mixed methods appraisal tool
PRISMA-P: Preferred reporting items for systematic review and meta-analysis protocols
SPIDER: Sample, phenomenon of interest, design, evaluation representing
