# Supplementary material for: Understanding the beliefs and attitudes towards mental health problems held by Muslim communities and acceptability of Cognitive Behavioral Therapy as a treatment: systematic review and thematic synthesis
Source: Discov Ment Health. 2023 Nov 23;3(1):26. doi: 10.1007/s44192-023-00053-2 (PMC10667170; doi:10.1007/s44192-023-00053-2)
Supplement: Supplementary file 1 — (PDF 389 KB) [file 44192_2023_53_MOESM1_ESM.pdf]

Additional file: Inclusion and Exclusion Criteria

	Inclusion	exclusion
Study Population.	Working age male and female adult Muslims that have not received psychological therapies for the treatment of mental health difficulties or only received treatment with CBT<br><br>On occasions, where the original paper did not state the religion of the participants studies conducted in countries where the population is above 90% Muslim (Afghanistan, Iran, Libya, Pakistan, Saudi Arabia, Somalia, Turkey) were included.	Studies focussing on children and adolescents, health care professionals, family and carers views were excluded.
Interventions.	Studies focussed on general barriers to accessing mental health services and were not limited to any specific psychological intervention	
Comparators.	Regardless of any comparator condition used in quantitative studies, all studies meeting inclusion criteria were included.	
Designs.	Study Qualitative, quantitative, questionnaire-based and mixed methods research designs were included.	
Outcomes.	Understanding of the barriers that prevent Muslims from accessing mental health services and barriers accessing CBT for mental health difficulties.	
